# The prognostic significance of chromosome 17 abnormalities in patients with myelodysplastic syndrome treated with 5‐azacytidine: Results from the Hellenic 5‐azacytidine registry

**DOI:** 10.1002/cam4.2090

**Published:** 2019-03-21

**Authors:** Panagiotis Diamantopoulos, Dafni Koumbi, Ioannis Kotsianidis, Vasiliki Pappa, Argiris Symeonidis, Athanasios Galanopoulos, Panagiotis Zikos, Helen A. Papadaki, Panayiotis Panayiotidis, Maria Dimou, Eleftheria Hatzimichael, George Vassilopoulos, Susan Delimpasis, Despoina Mparmparousi, Sotirios Papageorgiou, Eleni Variami, Marie‐Christine Kyrtsonis, Aekaterini Megalakaki, Maria Kotsopoulou, Panagiotis Repousis, Ioannis Adamopoulos, Flora Kontopidou, Dimitrios Christoulas, Alexandra Kourakli, Dimitrios Tsokanas, Menelaos Konstantinos Papoutselis, Georgios Kyriakakis, Nora‐Athina Viniou, the Hellenic MDS study group

**Affiliations:** ^1^ Hematology Unit, First Department of Internal Medicine Laikon General Hospital, National and Kapodistrian University of Athens Athens Greece; ^2^ Institute of Nuclear and Radiological Sciences National Center for Scientific Research Demokritos Athens Greece; ^3^ Department of Hematology University Hospital of Alexandroupolis Alexandroupoli Greece; ^4^ Haematology Division, Second Department of Internal Medicine Attikon General Hospital, National and Kapodistrian University of Athens Athens Greece; ^5^ Department of Internal Medicine University Hospital of Patras Rio Greece; ^6^ Department of Clinical Hematology 'G. Gennimatas' District General Hospital Athens Greece; ^7^ Department of Hematology 'St Andrew' General Hospital Patras Greece; ^8^ Haematology Laboratory, School of Medicine University of Crete Greece; ^9^ First Propedeutic Department of Internal Medicine, School of Medicine National and Kapodistrian University of Athens Athens Greece; ^10^ Department of Haematology University of Ioannina Ioannina Greece; ^11^ Department of Hematology Larissa University Hospital, University of Thessalia Larissa Greece; ^12^ Department of Hematology Evangelismos Hospital Athens Greece; ^13^ Hematology Laboratory Alexandra General Hospital Athens Greece; ^14^ Department of Hematology Metaxa Anticancer Hospital Piraeus Greece; ^15^ General Hospital of Kalamata Greece; ^16^ Second Department of Internal Medicine National and Kapodistrian University of Athens, Hippokratio General Hospital Athens Greece; ^17^ Department of Hematology 251 General Air‐Force Hospital Athens Greece

**Keywords:** 5‐azacytidine, chromosome 17 abnormality, myelodysplastic syndrome

## Abstract

In patients with myelodysplastic syndrome (MDS), the prognostic significance of chromosome 17 abnormalities has not yet been fully elucidated, except for isochromosome 17q that has been characterized as an intermediate risk abnormality in the Revised International Prognostic Scoring System (IPSS‐R). To further characterize the prognostic significance of chromosome 17 abnormalities we analyzed the hematologic and prognostic characteristics of 548 adult patients with MDS treated with 5‐azacytidine through the Hellenic 5‐azacytidine registry and found 32 patients with a chromosome 17 abnormality (6 with i[17q], 15 with ‐17, 3 with add[17p] and the rest with other rarer abnormalities, mostly translocations). The presence of a chromosome 17 abnormality was correlated with poor prognostic features (high IPSS, IPSS‐R, and WPSS scores) and a low overall survival rate (15.7 vs 36.4 months for patients without chromosome 17 abnormalities, Kaplan–Meier, Log Rank *P* < 0.00001), but these results were confounded by the fact that most (92.3%) of the cases with a chromosome 17 abnormality (with the exception of i(17q) that was found in all cases as an isolated abnormality) were found in the context of a complex karyotype. Nevertheless, one should not ignore the contribution of chromosome 17 abnormalities to the prognostic significance of a complex karyotype since 33.8% of complex karyotypes encompassed a chromosome 17 abnormality.

## 1 INTRODUCTION

Chromosome 17 abnormalities have been correlated with poor prognosis and treatment failure in several hematologic malignancies, but their role in myelodysplastic syndrome (MDS) has not yet been fully elucidated. Although isochromosome 17q i(17q) has been characterized as an intermediate risk cytogenetic abnormality in the Revised International Prognostic Scoring System (IPSS‐R) for MDS, the prognostic significance of monosomy 17 and of other structural chromosome 17 abnormalities has not yet been fully defined. In 2013, a study on the presence of chromosome 17 abnormalities in patients with MDS conferred valuable information on the characterization and prognostic implications of these cytogenetic abnormalities in a large cohort of patients with MDS, chronic myelomonocytic leukemia, and low‐blast acute myeloid leukemia (AML). The results of the study implied that chromosome 17 abnormalities have a great impact on the prognosis of patients with MDS.^1^

Among others, the prognostic significance of chromosome 17 aberrations may be correlated with the fact that they may lead to loss of 17p13.1 that encompasses the genetic locus of the tumor suppressor gene p53 (*TP53*). It is now evident that, although relatively uncommon, *TP53* mutations have a major impact οn the survival of patients with MDS.^2^, ^3^

The prognostic significance of chromosome 17 abnormalities in patients with MDS treated with hypomethylating agents has not been thoroughly studied. Information on the prognostic significance of chromosome 17 abnormalities in MDS seems to be highly valuable in the era of hypomethylating agents, especially since patients with MDS and AML with *TP53* mutations or a poor cytogenetic profile have been recently shown to have a favorable response to decitabine.^4^

The aim of the present retrospective study is to analyze the epidemiologic, hematologic, and prognostic characteristics of a large cohort of patients with MDS and chromosome 17 abnormalities treated with 5‐azacytidine. Data were collected through the Hellenic 5‐azacytidine registry. Through this data collection we primarily intended to study the prognosis of patients with chromosome 17 abnormalities and compare it to that of patients without such an abnormality. The role of chromosome 17 abnormalities as previously defined in large patient series may be altered by the use of hypomethylating agents. This study aims to define their prognostic role in a large series of patients treated with 5‐azacytidine. Results from the same registry on the prognostic significance of monosomal karyotype in MDS have been recently published.^5^

## 2 METHODS

### 2.1 Patients and methods

We retrospectively recorded through the Hellenic 5‐azacytidine registry the main demographic, hematologic, and treatment characteristics of adult patients with MDS treated with 5‐azacytidine monotherapy. Patient data from 28 centers meeting the 2016 WHO diagnostic criteria for MDS were recorded during a 7‐month period. This study was designed and conducted by the Hellenic MDS Study Group, a Scientific Group of the Hellenic Society of Hematology.

Patients with an evaluable karyotype at diagnosis were stratified according to the IPSS, the IPSS‐R, and the WHO classification‐based PSS (WPSS). Conventional routine cytogenetic analysis was performed at diagnosis, using standard protocols, as per common clinical practice. Cytogenetic results were interpreted and reported per the 2016 International System for Human Cytogenetic Nomenclature (ISCN, 2016). Cytogenetic abnormalities were classified into three groups according to the IPSS cytogenetic risk stratification or into five groups according to the IPSS‐R cytogenetic risk stratification. We isolated patients with chromosome 17 abnormalities and we further analyzed their hematologic and survival characteristics. We also carried out survival analyses of patients with chromosome 17 abnormalities in comparison to those with a complex (≥3 or ≥4 cytogenetic abnormalities) karyotype and with chromosome 7 abnormalities, due to the dismal prognosis correlated with these two cytogenetic profiles.

### 2.2 5‐azacytidine treatment

5‐azacytidine was administered as monotherapy subcutaneously (and in a minority of cases intravenously) at the approved dosing schedule by the US Food and Drug Administration and the European Medicines Agency of 75 mg m^−2^ d^−1^ for 7 consecutive days, every 28 days.

### 2.3 Response to treatment and survival assessment

Responses were assessed by an independent assessor, based on the modified 2006 International Working Group (IWG) response criteria for MDS. Patients achieving complete remission (CR), partial remission (PR), or hematological improvement (HI) were defined as responders, whereas patients with stable disease (SD) or failure (F) as nonresponders. Patients treated with at least one full cycle of treatment were considered evaluable for response. The overall survival (OS) rate was defined as the time interval from diagnosis to death from any cause.

### 2.4 Statistical analysis

IBM SPSS statistics, version 23.0 (IBM Corporation, North Castle, NY) was used for the statistical analysis of the results. The individual tests used are cited in the “Results” section, separately for each correlation.

## 3 RESULTS

Data from 632 patients with MDS treated with 5‐azacytidine were recorded. Eighty‐four patients without an evaluable karyotype at diagnosis were censored and data from 548 patients were eventually analyzed. The demographic, hematologic, and prognostic characteristics of the patients are shown in Table 1. Thirty‐two (5.8%) patients carried a chromosome 17 abnormality at diagnosis, as an isolated abnormality or in the context of a complex karyotype. Abnormalities of chromosome 17 were the seventh most common cytogenetic abnormality after complex karyotype, and abnormalities of chromosomes 7, 8, 3, 5, and 12, in frequency order. Detailed cytogenetic results are provided in Supplementary Table S1. Briefly, 24 (75.0%) cases were found in the context of a complex karyotype while all (6, 18.8%) cases of i(17q) were found as an isolated cytogenetic abnormality. Several translocations were also found between chromosome 17 and chromosomes 4, 7, 11 (2×), 19, and 21 (Table 2).

**Table 1:** Patients’ results

Characteristics	Patients without chromosome 17 abnormalities	Patients with chromosome 17 abnormalities	two‐sided *P*
Number of patients, N (%)	516 (94.2)	32 (5.8)	ΝΑ
Sex (male: female)	2.49	1.67	0.317
Age (at diagnosis), Median (range)	73.6 (43.9‐89.0)	71.0 (51.7‐86.6)	0.779
WHO 2016 (diagnosis), N (%)			0.598
MDS‐SLD	17 (3.3)	0 (0)	
MDS‐MLD	149 (28.9)	5 (15.6)	
MDS‐RS	19 (3.7)	1 (3.1)	
MDS with isolated del(5q)	4 (0.8)	0 (0)	
MDS‐EB‐1	139 (26.9)	10 (31.3)	
MDS‐EB‐2	188 (36.4)	16 (50.0)	
MDS with excess blasts (MDS‐EB)	327 (63.4)	26 (81.3)	0.055
Hb (g/dL), Median (range)	9.4 (3.5‐14.7)	8.9 (5.0‐11.2)	0.117
MCV (fl), Median (range)	95.0 (54.1‐122.9)	89.3 (56.0‐117.0)	0.004
Neutrophil count (×10^9^/L), Median (range)	1.32 (0.00‐3.40)	1.1 (0.2‐8.5)	0.411
Platelets (×10^9^/L), Median (range)	99.5 (1‐813)	100 (28‐800)	0.418
Peripheral blood blast percentage, N (%)	0 (0‐19)	0 (0‐10)	0.440
Cytopenias, N (%)			0.577
0	30 (5.8)	3 (9.4)	
1	157 (30.4)	6 (18.8)	
2	209 (40.5)	13 (40.6)	
3	120 (23.3)	10 (31.3)	
Bone marrow blast percentage, N (%)	7 (0‐20)	11 (1‐19)	0.019
IPSS‐R group			0.00001
Very low	20 (3.9)	0 (0)	
Low	103 (20.0)	0 (0)	
Intermediate	109 (21.1)	4 (12.5)	
High	174 (33.7)	7 (21.9)	
Very high	110 (21.3)	22 (65.6)	
Transfusion needs, N (%)	318 (61.6)	24 (75.0)	0.319
Ferritin (ng/mL), Median (range)	357 (3‐20750)	360 (117‐1271)	0.709
Age at 5‐azacytidine initiation (y), Median (range)	74.0 (44.1‐89.4)	73 (57‐85)	0.499
AML transformation, N (%)^a^	200 (38.8)	14 (43.7)	0.848
Response (IWG criteria), N (%)			0.398
F	115 (22.3)	8 (25.0)	
SD	146 (28.3)	4 (12.5)	
PR	69 (13.4)	5 (15.6)	
CR	75 (14.5)	7 (21.9)	
HI	86 (16.6)	6 (18.8)	
Lost to follow‐up	25 (4.8)	2 (6.3)	
Survival status (alive at data cut‐off), N (%)	298 (58.7)	21 (65.6)	0.456

AML, acute myeloid leukemia; CR, complete remission; EB, excess blasts; F, failure; HI, hematologic improvement.; IPSS‐R, Revised International Prognostic Scoring System; MDS, myelodysplastic syndrome; MLD, multilineage dysplasia; PR, partial remission; RS, ring sideroblast; SD, stable disease; SLD, single lineage dysplasia; WHO, World Health Organization.

a 47 patients lost to follow‐up, percentage applies on the remaining patients.

**Table 2:** Chromosome 17 abnormalities found by a conventional cytogenetic analysis at diagnosis in 548 patients with MDS treated with 5‐azacytidine

Abnormality	Number of patients, N (%)	Comments
All chromosome 17 abnormalities	32 (5.8)	
i(17)(q10)	6 (18.8)	All cases as isolated chromosomal abnormalities
‐17	15 (46.9)	All cases in the context of a complex karyotype; two cases with a concomitant translocation of chromosome 17; one case with add(17)(q23); one case with del(17)(q25)
add(17p11)	3	All cases in the context of a complex karyotype.
add(17p13)	1	
del(17)(q21)	1	
del(17)(p11.1)	1	
Translocation involving chromosome 17	6	All except two cases in the context of a complex karyotype; two cases with concomitant ‐17
dic(2‐17)	1	In the context of a complex karyotype

add, addition; del, deletion; dic, dicentric; i, isochromosome.

Patients bearing a chromosome 17 abnormality at diagnosis tended to be classified more often as MDS‐EB per the 2016 WHO classification of MDS than patients without a chromosome 17 abnormality (Pearson Chi‐Square two‐sided *P*, 0.055) and had a higher percentage of bone marrow blasts at diagnosis (11% vs 7%, Mann–Whitney *U* test, two‐sided *P*, 0.019). Moreover, patients with chromosome 17 abnormalities tended to be higher risk patients per all three prognostic scoring systems (Pearson Chi‐Square two‐sided *P*, <0.000001 for IPSS, IPSS‐R, and WPSS). These associations are highly predictable of course, since 75% of chromosome 17 abnormalities were found in the context of a complex karyotype, therefore corresponding to higher scores per the IPSS, IPSS‐R, and WPSS. No correlations were found between the remaining hematologic characteristics of the patients (neutrophil count, hemoglobin concentration, platelet count, peripheral blood blast count, number of cytopenias) and the presence of chromosome 17 abnormalities. A statistically significant correlation of chromosome 17 abnormalities with lower mean corpuscular volume (MCV) of the red blood cells (89.3 fL vs 95.0 for patients without chromosome 17 abnormalities, Independent‐Samples Mann–Whitney *U* test, two‐sided *P*, 0.005, Table 1) was noted, but the same finding was noted in patients with a complex karyotype as well.

The patients received a median of 8 (1‐66) cycles of 5‐azacytidine and at the time of data cut‐off 124 (22.6%) patients were still on treatment. Response to 5‐azacytidine did not differ between patients with and without chromosome 17 abnormalities (Pearson Chi‐Square two‐sided *P*, 0.189), while there was a marginally nonstatistically significant trend for shorter duration of response in patients with chromosome 17 abnormalities (5.4 vs 7.5 months, Kaplan–Meier, log Rank *P* = 0.053). Moreover, response to 5‐azacytidine did not differ between patients with an isolated chromosome 17 abnormality or a complex karyotype including a chromosome 17 abnormality (Pearson Chi‐Square two‐sided *P*, 0.392). Moreover, there was a nonstatistically significant trend for a higher CR rate in patients with chromosome 17 abnormalities compared to the remaining patients (21.9% vs 14.5%, Pearson Chi‐Square two‐sided *P*, 0.237). There was no association with the risk of transformation to AML for patients with and without chromosome 17 abnormalities (Pearson Chi‐Square two‐sided *P*, 0.482) or for patients with an isolated chromosome 17 abnormality or a complex karyotype including a chromosome 17 abnormality (Pearson Chi‐Square two‐sided *P*, 0.183), although in the whole group, the presence of a complex karyotype was correlated with higher risk of transformation to AML (Pearson Chi‐Square two‐sided *P*, 0.029).

In general, no statistically significant differences were found among patients with different abnormalities of chromosome 17 [namely ‐17, i(17q), add17, and translocations involving chromosome 17] as for the demographic, hematologic, and prognostic characteristics.

**Figure 1:**
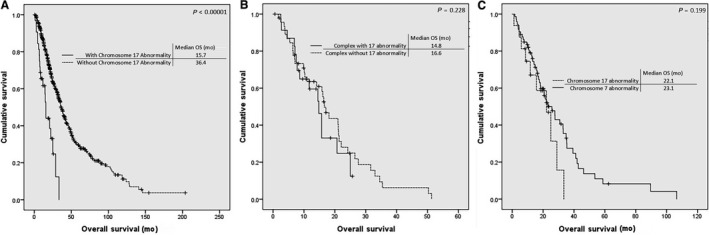
Kaplan–Meier curves for the overall survival (OS) of patients (A) with and without a chromosome 17 abnormality, (B) with a complex karyotype with and without a concurrent chromosome 17 abnormality, and (C) with chromosome 17 and chromosome 7 abnormalities

At the time of data cut‐off 240 (37.9%) patients were still alive and 10 (1.6%) were lost to follow‐up. The median follow‐up for survivors was 24.1 months. The median OS of the 548 patients was 35.3 months [95% Confidence Interval (CI), 31.9, 38.7]. The median OS of patients with chromosome 17 abnormalities was 15.7 months (95% CI, 14.0, 17.4) and was significantly lower than that of patients without chromosome 17 abnormalities (36.4 months, Log Rank *P* < 0.00001, Figure 1A). OS of patients with chromosome 17 abnormalities (with the exception of isochromosome 17q) was comparable to that of patients with a complex (≥3 or ≥4 lesions) karyotype. When comparing the OS of patients with a complex karyotype with and without a concomitant chromosome 17 abnormality, there was a nonstatistically significant trend for lower OS in patients with a complex karyotype encompassing a chromosome 17 abnormality (14.8 months vs 16.6 months, Kaplan–Meier, log Rank *P* = 0.228, Figure 1B). Moreover, since all cases with ‐17 were found in the context of a complex karyotype, a cox regression analysis with a model comprising two variables [(a) complex karyotype with or without ‐17 and (b) ‐17] revealed that the presence of ‐17 did not add to the dismal prognosis of the complex karyotype (HR, 1.010 [95% CI, 0.491‐2.074], *P* = 0.979). By multivariate analysis in a model comprising three variables (complex karyotype, presence of chromosome 17 abnormality, and presence of chromosome 7 abnormality), the presence of chromosome 17 abnormalities was not an independent prognostic factor for OS. Finally, patients with i(17q) that was found in all cases as an isolated abnormality had a median OS of 31.9 months that is comparable to the median OS of the whole group of 548 patients (35.3 months). Moreover, OS of patients with i(17q) was comparable to that of the rest of the patients with an intermediate cytogenetic risk per the IPSSR (31.9 months vs 32.5 months, Kaplan–Meier, log Rank *P* = 0.468).

## 4 DISCUSSION

Based on the data derived from the large national registry of patients with MDS treated with 5‐azacytidine, the most common cytogenetic abnormality of chromosome 17 is ‐17, followed by i(17q) and add17 while several uncommon translocations involving chromosome 17 follow. The frequency of chromosome 17 abnormalities in the present series (5.8%) is comparable to that presented in other large patient series ranging from 3.5% to 6.1%^1^, ^6^, ^7^, ^8^ and although they are relatively uncommon, they may be prognostically significant especially due to their correlation with loss of *TP53*. Their role as defined in large patient series may be altered by the use of hypomethylating agents.

The only abnormality of chromosome 17 that has already been characterized and used in the prognostic scoring systems for MDS is i(17q), defined as an intermediate cytogenetic risk lesion in the IPSS‐R I(17q) is relatively common in medulloblastoma, gastric, bladder, and breast cancer,^9^ and is frequently observed during progression of chronic myelogenous leukemia.^10^ In patients with MDS it is quite rare with an incidence of about 1%.^11^, ^12^ It has been correlated with male sex, mixed myelodysplastic and myeloproliferative characteristics, severe hypopigmentation of neutrophil nuclei, and high risk for progression to AML.^13^, ^14^ The present registry recorded six (1.1%) patients with isolated i(17q) that were predominantly male, had primarily MDS with excess blasts but no mixed myeloproliferative and myelodysplastic features (per definition, since only patients with a diagnosis of an MDS per the 2016 WHO classification were included in the analysis) nor a higher risk for progression to AML under treatment with 5‐azacytidine. Their median OS was comparable to that of the whole group. Another two patients with i(17)q were identified among the remaining patients of the registry with a diagnosis other than MDS per the 2016 classification. These two patients (not included in the analysis of patients with MDS) had a diagnosis of chronic myelomonocytic leukemia.

Patients with chromosome 17 abnormalities had lower OS than patients without chromosome 17 abnormalities, but this survival disadvantage can be attributed to the fact that, if we exclude i(17q) that was found only as an isolated abnormality, 92.3% of chromosome 17 abnormalities were found in the context of a complex karyotype. As shown in Table 2, most cases were correlated with a complex karyotype with more than four abnormalities, and in some cases with the concurrent presence of a chromosome 7 abnormality, thus bearing the poor prognosis attributed to these two groups of chromosomal abnormalities. Nevertheless, chromosome 17 abnormalities, especially ‐17, are a common component of complex karyotypes since, among 71 patients with a complex karyotype, 24 (33.8%) had a chromosome 17 abnormality. Hence chromosome 17 abnormalities may confer to the dismal prognosis of complex karyotypes. Moreover, the high percentage (33.8%) of complex karyotypes encompassing a chromosome 17 abnormality points out that the absence of *TP53* in patients with complex karyotypes with chromosome 17 abnormalities could be correlated with increased genetic instability conferred to the absence of *TP53*. Finally, there was a trend for lower OS in patients with chromosome 17 abnormalities when compared to complex karyotype. This trend should be interpreted bearing in mind that all the patients of this study were treated with a hypomethylating agent, thus they comprise a special population that has not been studied yet in terms of the prognostic significance of chromosome 17 abnormalities. The prognosis of patients with chromosome 17 abnormalities may be altered by the use of hypomethylating agents and this could explain why the already published results about the independent prognostic role of chromosome 17 abnormalities^1^ are not confirmed by our team. Moreover, recent data suggest that patients with *TP53* mutations show a favorable response to decitabine,^5^ implying that the use of hypomethylating agents could blunt the prognostic role of a poor cytogenetic or molecular profile. Therefore, it is possible that in our group the use of 5‐azacytidine may have altered the prognosis of patients with a complex karyotype and a chromosome 17 abnormality, blunting its additional adverse prognostic role. This hypothesis is reinforced by the fact that there was no difference in the response to 5‐azacytidine or the rate of AML transformation between patients with and without chromosome 17 abnormalities. Moreover, there was a nonstatistically significant trend for a higher CR rate in patients with a chromosome 17 abnormality that could be explained only if we consider a differential prognostic role of the use of hypomethylating agents in patients with chromosome 17 abnormalities.

Although 17p deletion was rare (1, <0.2%), all patients with i(17q) and ‐17 and many patients with translocations involving chromosome 17 lack at least a part of the short arm of chromosome 17, where *TP53* gene is located (17p13.1). The concurrent search for *TP53* mutations or 17p deletion by fluorescent in situ hybridization could significantly add to the characterization of patients with chromosome 17 abnormalities, but this is beyond the scopes of a registry. The fact that in our group, all cases of ‐17 were found in the context of a complex karyotype hinders further analysis. Nevertheless, in the study by Sanchez‐Castro et al, ‐17 was found to be an independent adverse prognostic factor after adjustment for the presence of a complex karyotype.

The association of chromosome 17 abnormalities with a lower MCV agrees with the already speculated prognostic significance of MCV for patients with MDS. Several authors have pointed out the significance of macrocytosis as a favorable prognostic factor in patients with MDS, especially in those with poor cytogenetic profiles.^15^, ^16^ Our study confirms this finding in patients with chromosome 17 abnormalities as well as in patients with a complex karyotype, since these groups of patients with dismal prognosis have a lower MCV compared to patients with a more favorable cytogenetic profile.

The retrospective nature of the study is an inherent limitation of this as well as other large studies on the prognostic significance of the cytogenetic profile of patients with MDS.^7^, ^8^ This has led to the lack of molecular results in the vast majority of patients and has also affected the completeness of data relevant to 5‐azacytidine toxicity and the disease course of the patients (subsequent treatments, allogeneic stem cell transplant) after discontinuation of treatment with 5‐azacytidine.

In conclusion, through the analysis of data from 32 patients with chromosome 17 abnormalities, among patients with MDS treated with 5‐azacytidine included in a large national registry, we identified the main hematologic and prognostic characteristics of the patients. Abnormalities of chromosome 17 are correlated with poor prognostic features (high IPSS, IPSS‐R and WPSS scores) and low overall survival. Response to treatment to 5‐azacytidine does not differ between patients with and without chromosome 17 abnormalities, while there is trend for shorter duration of response in patients carrying a chromosome 17 abnormality. Loss of 17p, mainly through ‐17, i(17q) and translocations involving chromosome 17, is correlated with poor prognosis, but these results are confounded by the fact that most cases of chromosome 17 abnormalities are found in the context of a complex karyotype. Nevertheless, one should not ignore the contribution of chromosome 17 abnormalities to the prognostic significance of a complex karyotype. Chromosome 17 abnormalities, although relatively rare, should not be overlooked when found, but further analysis of *TP53* loss or mutation should be carried out to further determine the prognosis of the patient. The potential differential impact of hypomethylating agents on patients with chromosome 17 abnormalities is highlighted here and should be further analyzed.
