# Supplementary material for: The prognostic significance of chromosome 17 abnormalities in patients with myelodysplastic syndrome treated with 5‐azacytidine: Results from the Hellenic 5‐azacytidine registry
Source: Cancer Med. 2019 Mar 21;8(5):2056–63. doi: 10.1002/cam4.2090 (PMC6536924; doi:10.1002/cam4.2090)
Supplement: Supplementary file 1 [file CAM4-8-2056-s001.docx]

Suppl. Table 1. Detailed cytogenetic results of all cases with a chromosome 17 abnormality.

| **Detailed cytogenetic results*** |
| --- |
| 46 XX, -3, del(5q)(q14q33), -7, +8, der(12)?inv(12)(p13;q24.1)**-17**, +mar1, +mar2 |
| 46,XX,del(5)(q13q33)[2]/46,sl,add(9)(q34)[2]/45,sl1,der(4)**t(4;17)(p16;q21)**,dic(7;9)(p11-p13;p11-p13), +8,-add(9)(q34),**-17**[4]/46,XX[1] |
| 40, XY, -2, der(3)t(3;?)(p?;?),del(5)(q13q33),-6,-7,+der(8)t(8;?)(p?;?),-9,-10, -11, -12, -16x2, **-17**x2, +20, -21x2, +mar1, +mar2, +mar3, +mar4, +mar5[5]/46,XY[5] |
| 78, ΧΧΥΥ, -1, -2, -3, -4, del(5)(q14q33), del(5)(q14q33), -6, der(6)t(6;?), del(7)(q22q34), -9, -11, -11, -12, -15, -16, **-17**, -18, -20, -20, -21, +r [11]/46,XX[7] |
| 43-46, XY, del(3)(p21), --4, -5, del(7)(q22q32), der(12)t(12;12)(p13;q24), der(12)t(4;12)(q12;p13), del(12)(q24), del(13)(q13q14), **-17**, add(19)(q13), -22, +1~4mar {18} /43, idem, add(2)(p21)[4]/46,XY[5] |
| 46,XY, del(5)(q31q35), **-17**, **add(17)(q23)**, +mar[4]/46,XY[15] |
| 43-45,XY, add(1)(p21), -5, del(7)(q22), der(11),**t(11;17)(q13;q11)**, **-17**, -18, -22, +1-3mar[6]/46,XY[18] |
| 46,XY, del(5)(q15q33) [4] / 44,XY, -3, del(5)(q15q33), der(11)t(3;11)(q21;p15), -12, der(13)t(12;13)(q15;q34), **-17**, +mar [7] / 45,XY, -3, del(5)(q15q33), del(6)(q13q21), -7, add(11)(p15), del(12)(p13), der(12)(p13), der(12)t(12;13)(q15;q34). der(13)t(12;13)(q15;q34), del(18)(q21), +mar[5]/ 46,XY[20] |
| 47, XY, del(5)(;13;33), +mar2[3] , 44, idem, -7, +11, der(11)t(11;?)(p?;?), -13, -14, **-17**, -19, -20, -21, der(21)t(?;p?), +mar1, +mar3, +mar4[7]/46,XY[20] |
| 45,X,-X,del(4)(q31q35),del(5)(q22q31),add(7)(q22),add(7)(q22),+?8,+10,del(11)(q22),  add(12)(p13),add(12)(p13),+?14,**del(17)(q25)**,**-17**,add(21)(p10),-21,-22[3] 46,XX[5] |
| 45XY,del(4),-7,**-17**,+mar[6]/46,XY[8] |
| 46,ΧΥ[9/20],46~47,ΧΥ,-4,-5,-6,add(9)(q34),-10,-11,-16,**-17**,+?22,+mar1,+mar2,+mar3,+mar4,+mar5,  +mar6,inc[11] 46,XX[9] |
| 43, XX, add(1)(p36), del(5)(q13q33), add(7)(p13), add(13)(q22), -16,**-17**, add(18)(p21), -19, add(19)(q13.1), -21, -22, +mar2, +mar3, +mar4[6]/ 46,XX[8] |
| 46,XY,del(5)(q13q33),del(6)(q23q27),add(8)(q24),add(9)(q22),add(11)(q25),**-17**,+r(11)(p15q25)[9]/ 46,XY[11] |
| 46,-48,XY,-5[22],del(7)(q11.2q23)[20],+del(8)(q23q24)[15],+11[18],add(16)(p13.3)[3],**-17**[20],-18[9],-20[20],add(21)(p11.2)[2],+22[2],+mar1[17],+mar2[15],+mar3[3],+mar4[cp22]/46,XY[2] |
| 45-47, XY, -3, del(5)(q22q32), **add(17)(p11)**, +1, +1~2 mar[cp7]/46,XY[3] |
| 45,X,-X,del(3)(p12),-5,-7,add(11)(q23),del(12)(p12),del(12)(q15q24),**add(17)(p11)**, +2mar[4/[ 46,XX[15] |
| 46,XX,-3,-5,+8,**add(17)(p11)**,i(21)(q10),+mar[10]/46,XX[5] |
| 42,add(X)(q2?4),Y,dic(3:16)(p21:?q24),-5,add(8)(p21),-15,**add(17)(p13)**,-18,add(22)(q13)[7]/41,sl,-12[3]/43,sl,add12p(13),+mar inc[3]/46,XY[7] |
| 44,XX,del(1)(p34p36),add(5)(q36),del(10)(q22q24),?**del(17)(q21)**,-18,-20[10]/44-45sl,add(10)(q26),-21,+mar1,+mar2, 2dmin [cp12]/46,XX[10] |
| 45,XX,del(5)(q31q34),-7,**del(17)(p11.1)**,-18,+mar[5]/45,XX,del(5)(q31q34),-7,+8,der(12)t(12:18)(p11:q11),**del(17)(p11.1)**,-18[15]/46,XY[5] |
| 46,XY, **t(17;19)(p13;p13.3)**[4]/ 46,XX[16] |
| 45,XYX,der(7)**t(7;17)(p22:q11.2)**,+11,-15,add(16)(q24),add(22)(p12)[5]/ 46,XX[15] |
| 44-43,Y,-X,t(3;9)(p13;q12),?del(5)(q13q31),add(9)(q34),der(11)t(11;15)(p14:q15),-15,**der(17)t(17;21)(p11.2;q11)**,-21[cp14]/46,XY[6] |
| 46,XX,del(5)(q14q31),**der(17)t(11;17)(q12;p13)**[11]/46,XX[5] |
| 43,XY,der **dic(2;17)(p25;p12)**ins(2;16)(p25;q11q22),del(5)(q13q31),der(7)del(7)(p15),del(7)(q22),  del(13)(q12q21,-16,-22[15]/46,XY[5] |
| *Detailed cytogenetic results of all cases with chromosome 17 abnormalities except for 6 cases with i(17q)(q10) that were found as an isolated chromosomal abnormality in all cases; chromosome 17 abnormalities are highlighted using bold style.  Abbreviations: i, isochromosome; add, addition; del, deletion; dic, dicentric; der, derivative; mar, marker; r, ring; ins, insertion; inv, inversion; dmin, double minute |
